# Epidemiology of Urban Canine Rabies, Santa Cruz, Bolivia, 1972–1997

**DOI:** 10.3201/eid0805.010302

**Published:** 2002-05

**Authors:** Marc-Alain Widdowson, Gustavo J. Morales, Sandra Chaves, James McGrane

**Affiliations:** * Unidad Nacional de Epidemiología Veterinaria, Santa Cruz, Bolivia; † Laboratorio de Investigación y Diagnóstico Veterinario, Santa Cruz, Bolivia; ‡ Rijksinstituut voor Volksgezonheid en Mileu (RIVM), Bilthoven, the Netherlands

**Keywords:** Epidemiology, rabies, Bolivia, urban health, risk factors, canine

## Abstract

We analyzed laboratory data from 1972 to 1997 from Santa Cruz, Bolivia, to determine risk factors for laboratory canine samples’ testing positive for *Rabies virus* (RABV). Of 9,803 samples, 50.7% tested positive for RABV; the number of cases and the percentage positive has dropped significantly since 1978. A 5- to 6-year cycle in rabies incidence was clearly apparent, though no seasonality was noted. Male dogs had significantly increased odds of testing positive for RABV (odds ratio [OR]=1.14), as did 1- to 2-year-old dogs (OR=1.73); younger and older dogs were at lower risk. Samples submitted from the poorer suburbs of the city were more likely to test positive for RABV (OR=1.71). We estimated the distribution of endemic canine rabies in an urban environment to facilitate control measures in a resource-poor environment.

Europe and North America have successfully controlled rabies in domestic animals, leaving wildlife as the main reservoir of concern (*1*,*2*). Nevertheless, rabies remains a serious public health hazard in many developing countries, where dog bites continue to be the main mode of transmission of the disease to humans. Throughout the world, an estimated 35,000 to 100,000 people a year die of rabies (*1*,*3*). The disease also elicits terror in communities, and subsequent control measures are drains on public health budgets (*4*,*5*). Rabies is a particular problem in the larger cities of less-developed countries, with sprawling, impoverished suburbs and high densities of dogs (*3*,*6*,*7*). Controlling rabies in urban dog populations is seen as a more cost-effective, long-term way to prevent human rabies than reliance on postexposure human treatment (*8*). To achieve control, knowledge of the epidemiology of rabies in dog populations has long been recognized as crucial (*9*).

In South America several larger urban areas have successfully eliminated rabies through legislation, education, and mass vaccination of dogs (*10*). Cities in poorer countries such as Bolivia, however, lag behind in control efforts, in large part because resources are scarce, and programs are poorly focused. In these situations, control efforts and resources must be more focused. For this, knowledge of risk factors for canine rabies in urban settings is needed to assess the danger to public health.

We analyzed 26 years of laboratory data on rabies diagnosis in dogs in the city of Santa Cruz, Bolivia, where rabies is endemic. We interpreted the results in light of possible biases to determine risk factors and temporal trends for rabies in the general dog population.

## Materials and Methods

### Study Area

Santa Cruz is a city of 1 million inhabitants, located in the department of Santa Cruz in the lowlands of eastern Bolivia. The city has been rapidly expanding at an average rate of 6.7 % per year since 1976 (*11*). The city center is circled by eight concentric ring-roads; municipal services and general socioeconomic status drop as distance increases from the city center. When we extrapolate from a study of the dog population conducted in 1996 (Laboratorio de Investigación y Diagnóstico Veterinario [LIDIVET], unpub. data), the canine population in 1999 in Santa Cruz was an estimated 276,034 dogs (1 dog per 4 inhabitants). Dogs are routinely vaccinated by private veterinarians and the municipality and Ministry of Health staff during 1- to 2-day annual public vaccination campaigns. Nevertheless, vaccination coverage data are unreliable. Additional municipal control measures for rabies include a dog pound, which collects and euthanizes up to 200 stray or aggressive dogs each month.

Canine rabies is a major problem in Santa Cruz, accounting for >90% of all animal rabies. In 1997, more than 2,178 people who had been bitten by dogs attended the municipal clinic for rabies prophylaxis; 1,464 required specific anti-rabies treatment. In 1997, three persons died of rabies, for an incidence of 0.30/10^5^ population, compared with the reported incidence of 0.025/10^5^ for Latin America in the same year (*3*). (Derived from 114 cases cited in that reference and using estimated Latin American population of 500 million.)

### Data Collection

LIDIVET receives samples of brain tissue for rabies diagnosis from suspected cases in animals and humans. For animal samples, species, age group, sex, and bite history have been noted since 1972. Since 1994, the location where the animal was found has also been recorded by ring-road.

Canine samples come from three sources: 1) dogs that have bitten people and have been killed or have died during the 10-day observation period; 2) dogs brought in by the public or private veterinarians because they showed suspicious symptoms; and 3) stray dogs routinely collected and euthanized by the pound. Impression smears of brain tissue from the cerebellum, Ammon's horn, and medulla are examined after staining by fluorescein-labeled anti-rabies globulin (Centocor, Malvern, PA).

### Data Analysis

All the records of canine samples examined at LIDIVET for rabies from within the municipal boundaries of Santa Cruz from 1972 through 1997 were analyzed. Secular trends were investigated with linear regression with EXCEL (Microsoft, Redmond, WA), using time in months as the independent variable (x) and number of cases per month as the dependent variable (y). A t-test was used to test for the significance of the slope (b). A centered moving average was applied to monthly numbers of positive cases to assess cyclicity in the data. To assess seasonality of rabies incidence, analysis of variance and the F-test were used to test for significant differences in the mean number of positives and mean percentage positive for each calendar month. Associations between numbers of positive samples and age group, sex, and ring-road were examined by the chi-square test for unequal odds and linear trend across groups (Epi-Info 6.04, Centers for Disease Control and Prevention, Atlanta, GA).

## Results

**Figure 1:**
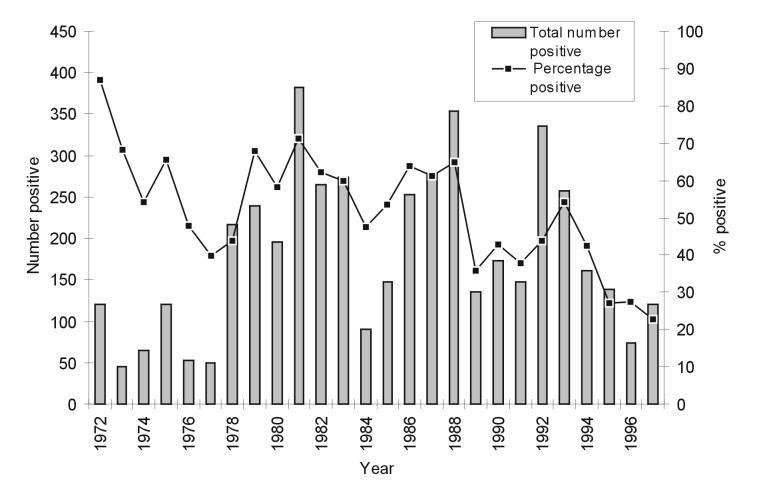
Percentage of rabies-positive samples and total number of positive samples* from dogs, Santa Cruz, Bolivia, 1972–1997. * By direct fluorescent antibody test.

From 1972 through 1997, 9,308 samples of canine brain tissue were analyzed in Santa Cruz; the annual number of tests varied from 66 to 764. Of all samples, 4,694 (50.4%) were positive for *Rabies virus* (RABV). The annual number of positive cases varied from 45 to 383, and the annual percentage of samples positive for RABV ranged from 22.81% to 87.05%. The annual percentage of positive samples dropped during the study period, as did the annual number of cases confirmed as positive; this trend was especially apparent in the last 5 years (Figure 1)

**Figure 2:**
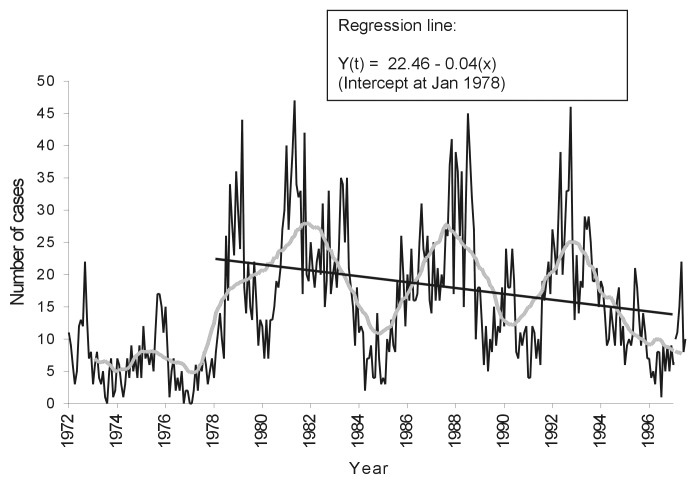
Positive rabies samples by month with a moving 24-month centered mean, Santa Cruz, Bolivia, 1972–1997, and regression line, 1978–1997.

The equation of the regression line was fitted only from 1978 because the low number of tests from 1972 through 1978 was uncharacteristic of the rest of the data. The number of monthly cases dropped significantly after 1978 (b = 0.04: t-test for slope= -4.53, 239 degrees of freedom [df], p<0.001) (Figure 2).

The cyclicity of rabies incidence suggested by Figure 1 is clearly shown by the 24-month centered moving average on cases per month in Figure 2. Since 1972, the mean number positive and mean percentage positive by month have shown no significant variation (F-test= 0.47, p=0.92).

### Gender

Twice as many samples and reported rabies cases were associated with male dogs (Table). The association between male dogs and sample positivity was significant, with an odds ratio [OR] of 1.14 (chi square=8.27, p=0.004).

**Table:** Canine laboratory samples examined for rabies and odds ratios for positive result, Santa Cruz, Bolivia, 1972–1997

Clinical and demographic characteristics	% of canine population^a^	No. of samples	No. (%) positive	OR (95% CI^b^)
Sex				
Female	50	2,969	1,419 (47.9)	1
Male	50	5,655	2,887 (51.1)	1.14 (1.04 to 1.25)
Unknown		684	368 (56.7)	
Age group				
<3 mo	20.6	1,624	713 (43.9)	1
3 mo to l yr	16.7	3,273	1,762 (53.8)	1.49 (1.32 to 1.68)
<l yr to 2 yrs	18.8	743	802 (57.5)	1.73 (1.49 to 2.00)
>2 yrs to 3 yrs	13.2	1,395	364 (49.0)	1.23 (1.03 to 1.47)
>3 yrs	30.7	1,317	463 (35.5)	0.69 (0.59 to 0.81)
Unknown		956	590 (61.7)	
Ring road^c^				
Within 4th	50.4	631	132 (20.9)	1
4th and beyond	49.6	488	152 (31.4)	1.71(1.29 to 2.26)
Unknown		8,189	4,410 (53.9)	
Total	100	9,308	4,694 (50.4)	

^a^ Percentages of total population of owned dogs (228,170) as estimated in 1996 (LIDIVET, unpub. data). ^b^ Confidence interval. ^c^ Data only available since 1994.

### Age

Of the 8,352 samples for which age was recorded, dogs <1 year old accounted for 4,893 (58.6%) samples and for 2,475 (60.3%) of 4,104 positive samples (Table). The odds for positive samples varied significantly between age groups (chi-square unequal odds = 189, p<0.001). Dogs 1 to 2 years of age were significantly more likely to test positive than dogs <3 months old (OR=1.73, chi square = 43, p<0.001). Testing positive for RABV was less likely in dogs >2 years of age, with dogs >3 years old at lower risk than those <3 months old (OR=0.69, chi square = 23, p<0.001).

### Location

The percentage of positive samples increased significantly as distance increased from the town center (chi-square trend = 25, 1 df p<0.001). This trend was especially evident after the sixth ring.

Despite similar estimated owned dog populations, fewer samples were received from outside the fourth ring than within. Samples from dogs outside the fourth ring were significantly more likely to test positive (Table; chi square = 14.67. 1 df, p<0.001).

## Discussion

The overall percentage of confirmed rabies in dogs submitted for diagnosis in Santa Cruz (50.4%) was similar to the 44% found in another urban study in Ghana (*12*). Other studies, not specifically urban, have shown percentages of samples positive varying from 54% to 67% (*13*–*15*). Canine rabies incidence appeared to decrease during the study period, especially since 1992. The significant drop in both number and percentage of positive samples suggested that this was not a reporting artifact. This decrease in incidence may be a result of vaccination, although vaccine coverage data are unreliable, and public sector vaccination has not been focused in recent years.

The data strongly suggested a 5- to 6-year cyclicity of rabies incidence; this cycle was most clearly apparent with a centered moving average of number of cases diagnosed per month. The cyclicity was independent of any changes in control measures and might explain the recent downturn in rabies cases. Some previous studies have reported a cyclical nature of rabies incidence (*14*,*16*), but this feature was not noted in an urban study in Delhi (*17*). Cyclicity is usually explained by increasing numbers of young, susceptible, unrestrained dogs in a population with low vaccine coverage. These factors lead to a drop in herd immunity, allowing rapid spread of the disease (*6*,*16*). However, even if underreporting is taken into account, the canine deaths from rabies in an epidemic year are unlikely to have substantially affected the number of susceptibles. A reduction in susceptibles may also be due to dogs’ becoming immune after recovering from clinical or inapparent infections. Recovery of dogs from rabies is well documented (*18*–*20*); one study showed 20% of experimentally infected dogs recovered (*21*). There is also serologic evidence that almost 20% of unvaccinated dogs in Thailand have been exposed to rabies (*22*).

Gender does seem to be a risk factor for sample positivity: male dogs had a significantly higher percentage of samples diagnosed positive (OR 1.14). This increased risk may be explained by males’ fighting over females. Studies in Mexico and India (*6*,*17*) show higher numbers of male dogs being affected, though the authors concluded that gender was not a risk factor, perhaps because of low numbers studied.

Rabies was not evenly distributed in Santa Cruz. The percentage of positive samples increased significantly with distance from the city center and as socioeconomic status dropped. In addition, a higher number of positives was reported from beyond the fourth ring-road, despite a similar-sized dog population. Some of the increase in percentage positive (but not in number positive) as distance increased from the center may be due to reporting bias. Fewer samples were submitted from outside the fourth ring, and these samples probably included a higher proportion of dogs that showed specific signs of rabies. An association of increased risk for rabies and low socioeconomic status has also been shown in Mexico (*6*). Lower vaccination coverage and increased densities of unrestrained dogs have previously been reported to be associated with poorer urban areas (*23*), a characteristic also shown in a recent survey of the canine population in Santa Cruz (LIDIVET, unpub. data).

Age was a clear risk factor for sample positivity in our study. The median age of a dog that tested positive for rabies was up to 1 year, as found in Mexico (*6*). The age group most at risk for testing positive for rabies, however, was 1- to 2-year-old dogs (OR=1.73). Dogs 3 months to 1 year of age were at intermediate risk (OR=1.49); however, this risk for rabies in dogs up to 1 year old may be underestimated. Perhaps because of the die-off of dogs of that age from all causes and the relative ease of carrying a puppy to the laboratory, the proportion of submissions (68% of all samples) from dogs <1 year was high, even relative to the population (37% of all dogs). This disproportion led to the finding of more positive samples but also to less specific reporting, with proportionally more nonrabid dogs with vague symptoms; such dogs would not have been submitted had they been older. This discrepancy may have decreased the percentage positive and thus underestimated the comparative risk for rabies in dogs <1 year old. It is nonetheless plausible that the risk for contracting rabies in dogs <1 year is lower than for 1- to 2-year-old dogs. Although dogs <1 year are less likely to have been vaccinated, sexually immature dogs are also less likely to roam, interact, and fight with other dogs. Puppies <3 months old may also benefit from passive immunity from their mothers. The large population of puppies, however, and their increased contact with children and adults make them a particular public health risk. The decreasing odds of sample positivity after 2 years of age may be due to increased likelihood of vaccination and less fighting among older dogs. Older dogs are also more likely to be owned.

We have shown that laboratory data can provide important information on risk groups and temporal trends for rabies in an urban environment. Especially if combined with additional work on the epidemiology of dog bites and seroepidemiologic studies, such data can help to effectively focus rabies-control efforts.
